# Comparison and identification of human coronary plaques with/without erosion using patient-specific optical coherence tomography-based fluid–structure interaction models: a pilot study

**DOI:** 10.1007/s10237-024-01906-7

**Published:** 2024-11-12

**Authors:** Yanwen Zhu, Chen Zhao, Zheyang Wu, Akiko Maehara, Dalin Tang, Liang Wang, Zhanqun Gao, Yishuo Xu, Rui Lv, Mengde Huang, Xiaoguo Zhang, Jian Zhu, Haibo Jia, Bo Yu, Minglong Chen, Gary S. Mintz

**Affiliations:** 1 School of Biological Science and Medical Engineering, Southeast University, Nanjing, 210096 China; 2 Department of Cardiology, The 2nd Affiliated Hospital of Harbin Medical University, National Key Laboratory of Frigid Zone Cardiovascular Diseases, Harbin, 150086 China; 3 The Key Laboratory of Myocardial Ischemia, Chinese Ministry of Education, Harbin, 150086 China; 4 Mathematical Sciences Department, Worcester Polytechnic Institute, Worcester, MA 01609 USA; 5 The Cardiovascular Research Foundation, Columbia University, New York, NY 10022 USA; 6 Department of Cardiac Surgery, Shandong Second Provincial General Hospital, Jinan, 250022 China; 7 Department of Cardiology, Zhongda Hospital, Southeast University, Nanjing, 210009 China; 8 First Affiliated Hospital, Nanjing Medical University, Nanjing, 210029 China

**Keywords:** Plaque erosion, Fluid–structure interaction, Vulnerable plaque, Optical coherence tomography, Erosion identification

## Abstract

Plaque erosion (PE) with secondary thrombosis is one of the key mechanisms of acute coronary syndrome (ACS) which often leads to drastic cardiovascular events. Identification and prediction of PE are of fundamental significance for disease diagnosis, prevention and treatment. In vivo optical coherence tomography (OCT) data of eight eroded plaques and eight non-eroded plaques were acquired to construct three-dimensional fluid–structure interaction models and obtain plaque biomechanical conditions for investigation. Plaque stenosis severity, plaque burden, plaque wall stress (PWS) and strain (PWSn), flow shear stress (FSS), and ΔFSS (FSS variation in time) were extracted for comparison and prediction. A logistic regression model was used to predict plaque erosion. Our results indicated that the combination of mean PWS and mean ΔFSS gave best prediction (AUC = 0.866, 90% confidence interval (0.717, 1.0)). The best single predictor was max ΔFSS (AUC = 0.819, 90% confidence interval (0.624, 1.0)). The average of maximum FSS values from eroded plaques was 76% higher than that from the non-eroded plaques (127.96 vs. 72.69 dyn/cm^2^) while the average of mean FSS from erosion sites of the eight eroded plaques was 48.6% higher than that from sites without erosion (71.52 vs. 48.11 dyn/cm^2^). The average of mean PWS from plaques with erosion was 22.83% lower than that for plaques without erosion (83.2 kPa vs. 107.8 kPa). This pilot study suggested that combining plaque stress, strain and flow shear stress could help better identify patients with potential plaque erosion, enabling possible early intervention therapy. Further studies are needed to validate our findings.

## Introduction

Acute coronary syndrome (ACS) is the main cause of death in patients with coronary heart disease. It is generally accepted that atherosclerotic plaque rupture (PR) or plaque erosion (PE) is the main pathological basis of most ACS. Approximately 65% of acute coronary syndromes are caused by plaque rupture, while 25%–30% of acute coronary syndromes are caused by plaque erosion (Fahed and Jang 2021; Kim et al. 2020; Kolte et al. 2021; Virmani et al. 2006; White et al. 2016). Most vulnerable plaque research has been focused on plaque rupture due to its drastic clinical consequences. Plaque erosion and related mechanisms have been under-investigated. Due to the differences in their development and process, PE may have different pathological and clinical characteristics from plaque rupture, and should be carefully investigated.

Plaque erosion usually starts from the apoptosis and abscission of vascular endothelial cells. Due to the absence of endothelial cells, collagen fibers activate platelets and accumulate in eroded plaques, then forming large volume of thrombosis which can cause vascular occlusion, leading to drastic or even fatal cardiovascular events. Previous studies show that patients with plaque erosion are common in young patients and patients with non-ST segment elevation acute coronary syndrome (NSTE-ACS) (Luo et al. 2021). Compared with the ruptured plaque, the stenosis of the lumen caused by the eroded plaque is less severe, and the thrombus formed in the lumen is mainly white thrombus (Fang et al. 2022; Virmani et al. 2000). Although progresses have been made in the morphological and pathophysiological characteristics of plaque rupture and plaque erosion, both biomechanical mechanisms contributing to and associated with plaque erosion, and differences between the biomechanical characteristics of plaques with and without erosion are not well understood.

Optical coherence tomography (OCT), as an emerging imaging modality with a resolution of 10–20 μm, can help to evaluate the morphology of atherosclerotic plaque in vivo. However, OCT cannot directly visualize the endothelium. Therefore, although endothelial cells damage is a pathological characteristic of plaque erosion, the absence of endothelial cells cannot be used as an OCT standard to identify it. Jia and co-workers proposed a classification method based on the characteristics of criminal lesions in ACS patients observed by OCT in vivo (Jia et al. 2013). Using their classification, we can discriminate between non-eroded plaques and eroded plaques. Based on their OCT data and classifications, patient-specific fluid structure interaction (FSI) models can be constructed for non-eroded and eroded plaques to investigate their biomechanical behaviors for a better understanding of biomechanical mechanisms associated with plaque erosion.

To date, computational models based on OCT images have gradually become a common tool to study the biomechanical mechanisms of plaque erosion, especially computational fluid dynamics (CFD) models (Russo et al. 2023; Weng et al. 2023). Based on the hypothesis that eroded plaques begin with the shedding and apoptosis of endothelial cells by force, some researchers focused on flow environment of plaque erosion (Campbell et al. 2013; McElroy et al. 2021; Thondapu et al. 2021). To determine the flow environment permissive for PE, McElroy and co-workers constructed CFD models to analyze the lumen geometry of 17 patients with OCT-defined PE and found that the majority of OCT-defined erosions occur where the endothelium is exposed to elevated flow (McElroy et al. 2021). To investigate whether local hemodynamics correlate with the site of plaque erosion, Campbell et al. constructed a three-dimensional patient-specific model of the coronary arteries, simulated pulsatile blood flow using CFD methods, calculated flow wall shear stress (FWSS) and oscillatory shear index (OSI), and studied the anatomical characteristics of the plaque erosion site by examining branching and local curvature in postmortem cardiac angiograms (Campbell et al. 2013). The results demonstrated that high magnitude wall shear stress is not necessary for erosion to occur. To investigate the local hemodynamic differences between plaque rupture and plaque erosion, Thondapu et al. performed intracoronary OCT imaging in patients with ACS caused by plaque rupture and plaque erosion, and constructed three-dimensional CFD model to compare the results of effective shear stress (ESS), effective shear stress gradient (ESSG) and oscillatory shear index (OSI), which showed that ESSG and OSI play a key role in plaque rupture and erosion, respectively (Thondapu et al. 2021). All the above investigations used CFD models. In the current literature, solid mechanics mechanism of plaque erosion is far less studied. In 2021, Wang et al. constructed 3D patient-specific FSI models with different thrombus volumes, fluid-only, and structure-only models to simulate and compare the biomechanics under different clinical situations (Wang et al. 2021) and they found residual thrombus would elevate wall shear stress and intracoronary thrombus might influence coronary hemodynamics and solid mechanics differently.

In this paper, we continue our plaque erosion investigation from both flow and solid mechanics sides using OCT-based FSI models. OCT coronary plaque data from 16 patients (eight plaques with erosion, eight plaques without erosion) were obtained and patient-specific FSI models were constructed to investigate the biomechanical mechanisms contributing to plaque erosion. Biomechanical differences between eroded and non-eroded plaques and morphological and biomechanical factors to identify plaques with and without erosion were carefully examined.

## Data, models and methods

### Optical coherence tomography and angiography data acquisition and processing

This study is a retrospective study. 16 patients, including eight patients with erosion and eight patients without erosion, were randomly selected from the existing patient pool. De-identified existing in vivo OCT and angiography data were obtained from patients with coronary heart diseases at Cardiovascular Research Foundation (CRF), the Second Affiliated Hospital of Harbin Medical University and Zhongda Hospital Southeast University. Data were acquired using protocol approved by the local institute and informed consent was obtained. OCT images were acquired with ILUMIEN OPTIS System, and Dragonfly or Dragonfly JP Imaging Catheter (St. Jude Medical, Westford, Massachusetts). Plaque erosion on OCT images was defined as 1) plaque with intact fibrous cap (no fibrous cap disruption) with thrombosis, or 2) plaque with intact fibrous cap with no thrombosis, but irregular luminal surface was present, or 3) plaque with thrombus to obscure the underlying plaque structure, and no superficial lipid pool and calcification were found proximal or distal to the thrombus (Jia et al. 2013). During the selection process, patients with balloon pre-dilation, stent thrombosis or neoatherosclerosis, massive thrombosis, and poor image quality were excluded; and ACS due to plaque rupture, calcified nodules, coronary hairpin layer, severe stenosis, coronary spasm, difficulty in judging the nature of lesions, or other pathogenic factors were also excluded. After pre-screening, 16 patients were selected from 30 patients, with stent thrombosis (6) and poor image quality (8) excluded. Patient demographic data are shown in Table 1.

**Table 1:** Patients demographic and plaque information

Patient	Age	Gender	BP (mmHg)	Vessel	Plaque classification
P1	64	M	126/83	RCA	Erosion
P2	58	M	117/73	LAD	Erosion
P3	69	F	150/80	RCA	Erosion
P4	64	M	105/72	LAD	Erosion
P5	42	M	110/60	LAD	Erosion
P6	41	M	97/56	LAD	Erosion
P7	55	M	110/70	LAD	Erosion
P8	46	M	136/88	LAD	Erosion
P9	81	F	138/71	RCA	No Erosion
P10	72	M	143/80	LCX	No Erosion
P11	80	F	136/89	LCX	No Erosion
P12	80	F	136/89	RCA	No Erosion
P13	66	F	172/65	RCA	No Erosion
P14	73	M	150/55	LCX	No Erosion
P15	63	F	138/82	LAD	No Erosion
P16	61	M	128/78	LCX	No Erosion

BP = Blood pressure; M = Male; F = Female; RCA = Right coronary artery; LAD = Left anterior descending; LCX = Left circumflex

More complete patient lab results and clinical history are given in Table 2. There are statistically significant differences in hypertension, cholesterol level, and low density lipoprotein between the two groups. Differences in dyslipidemia, high density lipoprotein, triglycerides and smoking between the two groups were not statistically significant.

**Table 2:** Lab results and clinical history comparison for plaques with and without erosion

Plaque Type	Patient	HT	DM	Cholesterol (mg/dL)	HDL (mg/dL)	LDL (mg/dL)	Triglycerides (mg/dL)	Smoking
Plaque with Erosion	P1	0	1	82.08	22.86	40.32	42.12	1
	P2	0	1	109.08	23.94	68.94	39.42	1
	P3	0	0	86.04	25.20	53.46	17.46	0
	P4	0	1	192.64	46.51	133.98	53.10	0
	P5	0	1	99.03	92.90	84.30	28.32	1
	P6	0	–	–	–	–	–	0
	P7	0	0	135.73	56.46	76.18	94.77	0
	P8	0	1	89.19	66.45	64.98	153.54	1
Plaque without Erosion	P9	1	0	173.00	60.00	89.00	30.00	0
	P10	1	1	163.00	45.00	97.00	117.00	1
	P11	0	0	176.00	43.00	101.00	170.00	0
	P12	0	1	225.00	59.00	131.00	254.00	0
	P13	0	0	169.00	56.00	101.00	73.00	0
	P14	1	1	189.00	57.00	103.00	122.00	0
	P15	1	1	177.00	68.00	86.00	135.00	0
	P16	1	1	129.00	41.00	71.00	128.00	0
*P value*		0.0256	1	0.0205	0.5358	0.0373	0.0541	0.2821

HT = hypertension. 1 represents patients with hypertension and 0 represents patients without hypertension. DM = dyslipidemia. 1 represents patients with dyslipidemia and 0 represents patients without dyslipidemia. HDL = high density lipoprotein; LDL = low density lipoprotein. The unit of cholesterol, HDL, LDL, and triglycerides is mg/dL. For Smoking, 1 represents patients with smoking history and 0 represents patients without smoking history

OCT images were manually segmented by experts to obtain the contours of atherosclerotic plaque components and thrombi. Details were published previously (Guo et al. 2019; Lv et al. 2020, 2023). Briefly, the plaque components were segmented into three classifications: lipid core, calcification and fibrous tissue, where lipid tissue was identified as homogeneously signal-poor regions with the diffuse border, calcification tissue as low signal region with the sharp boundary and fibrous tissues as rich and homogeneous high signal areas. White thrombi (red thrombi were not considered because signal-free shadow shaded the plaque behind red thrombi) showed signal-rich, low-backscattering protrusions in the OCT image. Lumen and media-adventitia boundary (external elastic membrane, EEM, defined as outer-boundary) were segmented and saved for model construction. The segmentation of plaque components was performed by two investigators who were blinded to the segmentation results by the other investigator. For cases with different segmentations (error > 10%), the segmentations were performed again by a senior investigator who was blinded to the segmentation results from the two investigators. The final results were then determined by the senior investigator. The senior investigator also reviewed cases with errors < 10% and selected one out of the two based on his experiences for model construction. The accuracy of a segmented contour (lumen, wall, lipid, thrombus, or CA) was defined as the ratio of the intersection of the areas bounded by the contours from the two investigators (this is the agreed area) over the union of the two areas.

**Fig. 1:**
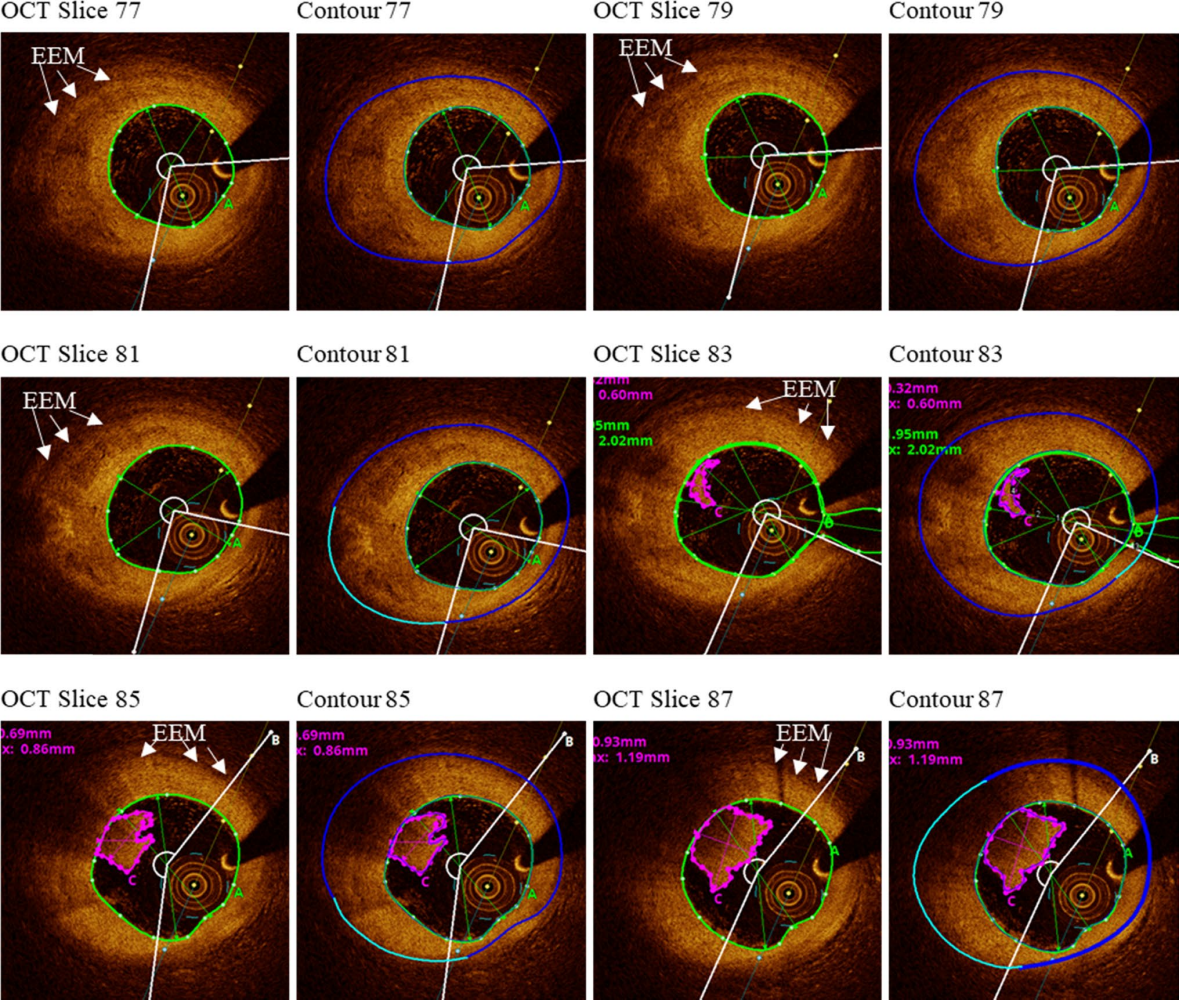
Selected OCT slices with segmented contours showing the determination of slice out-boundary using OCT images. EEM: external elastic membrane (see arrows in OCT slices). The part of the contour (shown with light blue) was determined using the contours of neighboring slices as references based on the assumption that the outer-boundary would change continuously and the wall thickness of adjacent slices would be similar. Green line: lumen contour; Blue line: vessel outer-boundary; Magenta line: thrombus contour

**Fig. 2:**
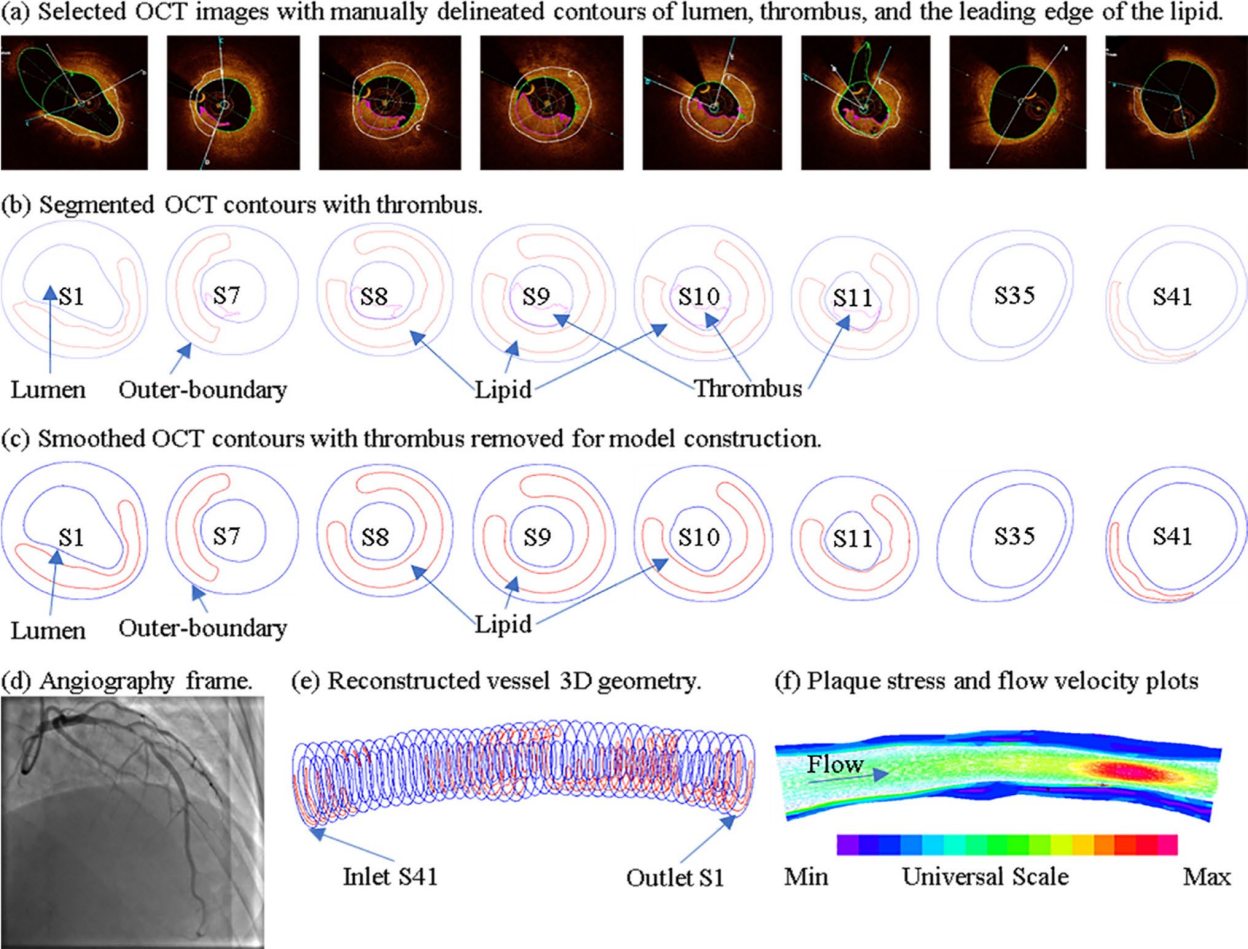
Selected OCT images, segmented contours with and without thrombus, angiography, reconstructed 3D coronary geometry, plaque stress and flow velocity plots. Green line: lumen contour; Blue line: vessel outer-boundary; White line: cap outline between lumen and lipid; Magenta line: thrombus contour

Figure 1 shows selected OCT slices with segmented contours to explain the determination of slice outer-boundary using OCT images. For those slices whose external elastic membrane could be clearly observed, the contours were segmented directly. For the part where the outer-boundary could not be detected in OCT image due to intima thickening and imaging artifacts, it was determined using the contours of neighboring slices based on the assumption that the outer-boundary would change continuously and the wall thickness of adjacent slices would be similar. Consideration was also given so that the entire outer-boundary contour was consistent for the whole slice. Once the outer-boundary was settled, the trailing edge of the lipid contour was determined by assuming the lipid core occupied two-thirds of the space behind the leading edge of lipid contour (Wang et al. 2021). The segmented plaque lumen, component contours and outer-boundary were saved for model construction. X-ray angiographic data were used to get location of the coronary artery vessel segment, stenosis severity and vessel curvature. Vessel centerline was obtained from X-ray angiography using ImageJ v1.54 h software. Co-registration of OCT and angiographic data were performed to reconstruct the 3D coronary geometry. The segmented slices were placed along the centerline keeping their cross-sections perpendicular to the center line, with slice distance set to the distance of OCT frames (0.2 cm). In 3D geometry reconstruction, axial and circumferential smoothing were performed using linear interpolation to remove discontinuities and extreme irregular geometries so that generating finite element mesh generation became possible. Figure 2 shows sample OCT slices, their segmented contours and the reconstructed vessel 3D geometry using angiography data.

Original OCT images included thrombi which have considerable impact on plaque wall stress/strain and flow behaviors (Wang et al. 2021). However, our purpose is to seek mechanisms leading to erosion. It would be more reasonable to “go back to history” and find plaque biomechanical conditions at its pre-erosion stage. To do this, thrombi were removed from segmented OCT contours and the smoothed contours (see Fig. 2c) were used to construct models and obtain biomechanical characteristics for our investigations.

### The 3D fluid–structure interaction (FSI) model

**Fig. 3:**
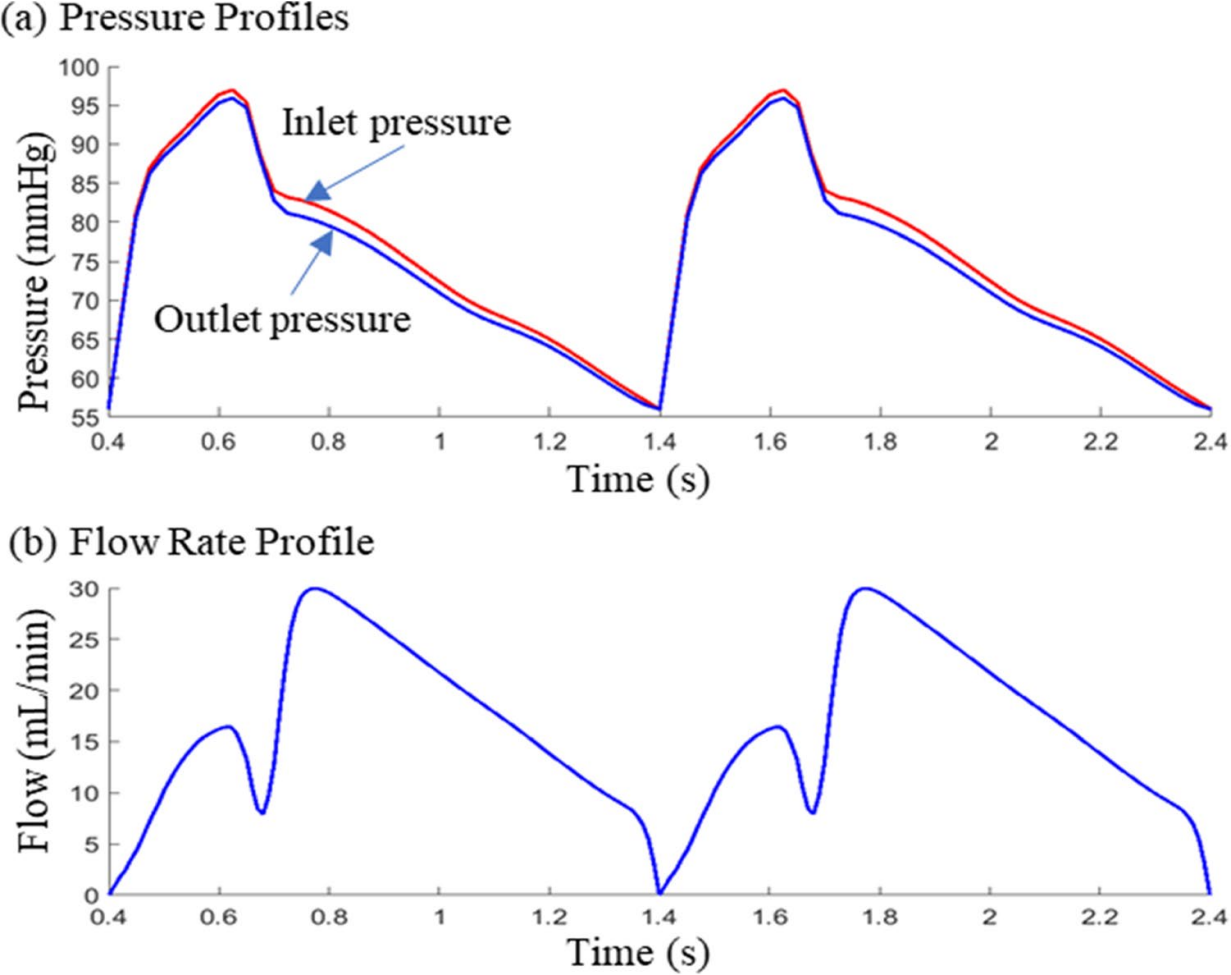
Inlet and outlet pressure conditions and flow profiles from a sample patient

Most previous publications used CFD models which missed the solid mechanics part and could not give complete picture for plaque biomechanical environment. In this paper, fully-coupled 3D FSI models were used to include both flow and solid mechanics for our study. For the fluid part, blood was assumed to be laminar, viscous, incompressible and Newtonian. Patient-specific arm pressure values were used to prorate pressure profile in the literature to generate inlet pressure curve. The outlet pressure profile was numerically adjusted to produce coronary flow profile (prorated from the literature) and match the flow rate obtained from angiography data. Figure 3 shows the inlet/outlet pressure and flow profiles from a sample patient. No-slip boundary conditions and force balance were imposed on the blood–vessel interfaces. The arbitrary Lagrangian–Eulerian description were adopted to handle the free-moving boundary (the interface). The flow model and boundary conditions are given below:

1 $$ \rho \left( {\frac{\partial u}{{\partial t}} + \left( {\left( {u - u_{g} } \right) \cdot \nabla } \right)u} \right) = - \nabla p + \mu \nabla^{2} u, $$

2 $$ \nabla \cdot u = 0, $$

3 $$ {\uprho }\frac{{\partial_{{v_{i} }}^{2} }}{{\partial t^{2} }} = \frac{{\partial \sigma_{ij} }}{{\partial x_{j} }},\;i,j = { 1},{ 2},{ 3}, $$

4 $$ \varepsilon_{ij} = \frac{1}{2}\left( {\frac{{\partial v_{j} }}{{\partial a_{i} }} + \frac{{\partial v_{i} }}{{\partial a_{j} }} + \frac{{\partial v_{\alpha } }}{{\partial a_{i} }}\frac{{\partial v_{\alpha } }}{{\partial a_{j} }}} \right),\;{\text{i , j = 1, 2, 3,}} $$

5 $$ \sigma_{ij} = \frac{1}{2}\left( {\frac{\partial W}{{\partial \varepsilon_{ij} }} + \frac{\partial W}{{\partial \varepsilon_{ji} }}} \right), $$

6 $$ \sigma_{ij} \cdot n_{j} |_{{{\text{outer}} - {\text{boundary}}}} = 0, $$

7 $$ \sigma_{ij}^{r} \cdot n_{j} |_{{{\text{interface}}}} = \sigma_{ij}^{s} \cdot n_{j} |_{{{\text{interface}}}} , $$

8 $$ u|_{{\Gamma }} = \frac{\partial v}{{\partial t}};\;\frac{{\partial {\text{u}}}}{{\partial {\text{n}}}}|_{{{\text{inlet}},{\text{ outlet}}}} = 0, $$

9 $$ p|_{{{\text{inlet}}}} = p_{{{\text{in}}}} \left( t \right);\;p|_{{{\text{outlet}}}} = p_{{{\text{out}}}} \left( t \right), $$

where, *u* and *p* are fluid velocity and pressure, *μ* is the dynamic viscosity, *u*_*g*_ is the mesh velocity, Г represents blood–vessel or blood–thrombus interfaces, *σ* is the stress tensor (superscripts indicate different materials), *ε* is the strain tensor, *n* is normal vector and *v* is the solid displacement vector.

Coronary vessel tissue was assumed to be hyperelastic, anisotropic, nearly incompressible, and homogeneous. A modified Mooney-Rivlin material model was used to describe the material properties of coronary vessel tissue, and the strain energy density function is shown as below (Guo et al. 2021, 2017; Holzapfel et al. 2000):

10 $$ \begin{aligned} {\text{W}} = & c_{1} \left( {I_{1} - 3} \right) + c_{2} \left( {I_{2} - 3} \right) + D_{1} \left[ {{\text{exp}}\left( {D_{2} \left( {I_{1} - 3} \right)} \right) - 1} \right] \\ & + \left( {K_{1} /K_{2} } \right)\left\{ {{\text{exp}}\left[ {K_{2} \left( {I_{4} - 1} \right)^{2} } \right] - 1} \right\}, \\ \end{aligned} $$

11 $$ I_{1} = \sum C_{ij} ; I_{2} = 1/2\left[ {I_{1}^{2} - C_{ij} C_{ij} } \right], $$

where, *C* = *[C*_*ij*_*]* = *X*^*T*^*X* is right Cauchy-Green deformation tensor. *X* = *[X*_*ij*_*]* = $\partial {x}_{i}/\partial {a}_{j}$, (*a*_*j*_) is the original position, (*x*_*i*_) is the current position. *I*_*1*_ and *I*_*2*_ are the first and second invariants of *C*. *C*_*i*_*, D*_*i*_*, K*_*1*_ and *K*_*2*_ are material parameters, *I*_*4*_ = *C*_*ij*_*(n*_*c*_*)*_*i*_*(n*_*c*_*)*_*j*_*,* and *n*_*c*_ is the unit vector in the circumferential direction of the vessel.

Plaque components (lipid and calcification) were assumed to be hyperelastic, isotropic, nearly incompressible and homogeneous, and the strain energy density function is shown as below:

12 $$ W_{{{\text{iso}}}} = c_{1} \left( {I_{1} - 3} \right) + c_{2} \left( {I_{2} - 3} \right) + D_{1} \left[ {{\text{exp}}\left( {D_{2} \left( {I_{1} - 3} \right)} \right) - 1} \right], $$

**Fig. 4:**
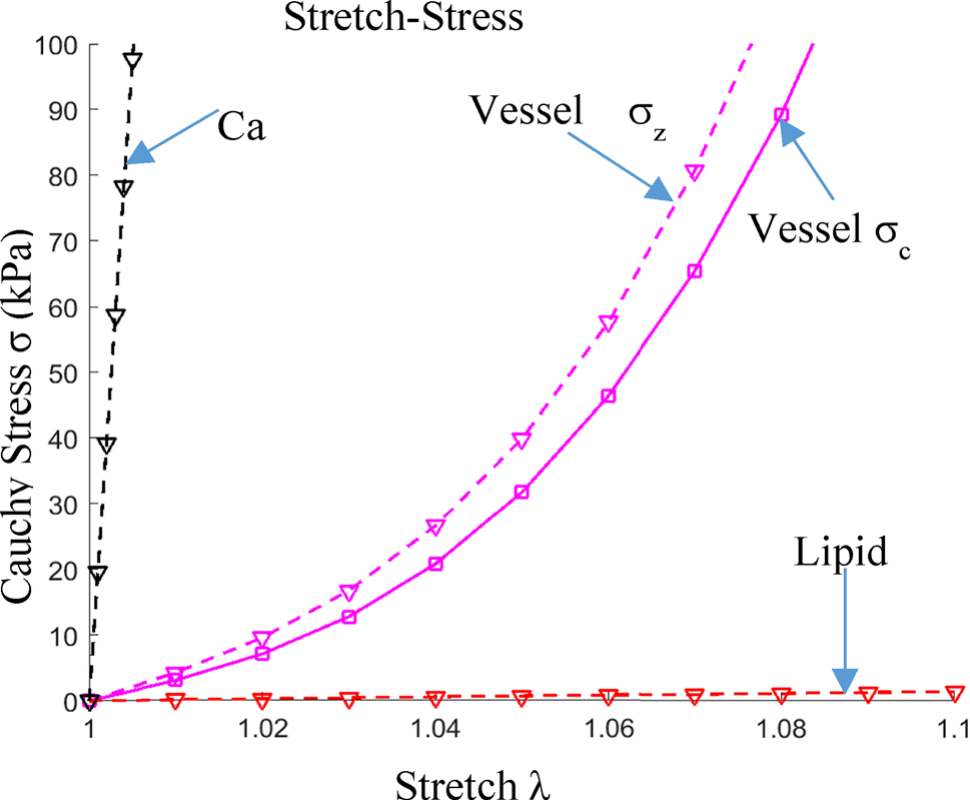
Stress-stretch curves derived from Mooney-Rivlin models of vessel, lipid and calcification used in finite element modeling. σ_c_: Circumferential stress; σ_z_: Axial stress

The material parameters used in this paper are shown in Table 3. The values are consistent with the data available in the literature and the material curves are shown in Fig. 4 (Guo et al. 2021, 2017; Holzapfel et al. 2000).

**Table 3:** Parameter values for material models used in the study

Tissue type	C1(kPa)	C2(kPa)	D1(kPa)	D2	K1(kPa)	K2
Vessel	− 515.6	45.05	247.3	2.0	14.1	23.5
Lipid	0.5	0	0.5	1.5		
Ca	920	0	360	2.0

### Model construction process and solution methods

Patient-specific three-dimensional FSI models based on OCT data (thrombi removed) were constructed with preshrink-stretch process applied. With the centerline obtained by X-ray angiographic data, segmented OCT slices were placed on the centerline to construct 3D coronary geometry obtained by co-registration of OCT and angiographic data (see Fig. 2). Since OCT images were obtained under in vivo conditions and coronary arteries were subjected to blood pressure and axially stretch, a preshrink-stretch process with axial and circumferential shrink was applied to in vivo OCT slices to obtain zero-load state of the vessel segment. Axial stretch and pressure conditions were then applied to the zero-load state so that the vessel segment would recover its in vivo morphology with proper stress/strain conditions. Axial shrinkage for each slice was assumed to be 5% for all plaques while circumferential shrinkage rate was determined iteratively to match its in vivo morphology. More details of the preshrink-stretch process could be found in (Huang et al. 2016; Tang et al. 2014; Wang et al. 2019).

The 3D FSI models were solved by the finite element software ADINA 9.6 (Adina R & D, Watertown, MA, United States) following our established procedures (Guo et al. 2019). Nonlinear incremental iterative procedures were used to solve the models. Hexahedral mesh was used for all solid models, and tetrahedral mesh was used for all fluid models. The models for three cardiac cycles were calculated and the data of the last cycle was used as the results for analysis. Mesh analysis was performed by refining mesh density by 10% until changes of solutions became less than 2% (Guo et al. 2019). For the 16 models, the average total solid model element number was 60258, ranging from 25200 to 95040; the average total fluid model element number was 85660, ranging from 29288 to 305801.

### Data extraction and processing

The FSI models for plaques with and without erosion were solved and results were extracted from model solutions for comparison. Three morphological characteristics (plaque stenosis by area, plaque burden, and cap thickness) and three biomechanical conditions (plaque wall stress, plaque wall strain and flow shear stress) were compared. Plaque stenosis by area was defined as:

13 $$ {\text{Plaque stenosis by area }} = { 1 }{-}{\text{ min lumen area }}/{\text{ reference lumen area}}, $$

where reference lumen area is the lumen area at the proximal site of the lesion (Mintz et al. 2001; Prati et al. 2010). Plaque burden was calculated as below:

14 $$ {\text{Plaque burden }} = { 1 }{-}{\text{ lumen area }}/{\text{ wall area}}, $$

where wall area is the area inside the vessel outer-boundary.

Results of plaque maximum principal stress/strain (plaque stress/strain) and flow shear stress (FSS) at the interface corresponding to the maximum blood pressure from each plaque were extracted. Following the commonly used terms, plaque wall stress (PWS), strain (PWSn) and flow wall shear stress (FSS) were used in this paper. Flow wall shear stress is defined as the value of the flow maximum principal shear stress on the lumen wall. To evaluate the temporal change in mechanical metrics, ΔFSS is defined as the difference of FSS between maximum and minimum pressure conditions:

15 $$ \Delta {\text{FSS}} = {\text{FSS}}_{{{\text{maxP}}}} - {\text{FSS}}_{{{\text{minP}}}} $$

**Fig. 5:**
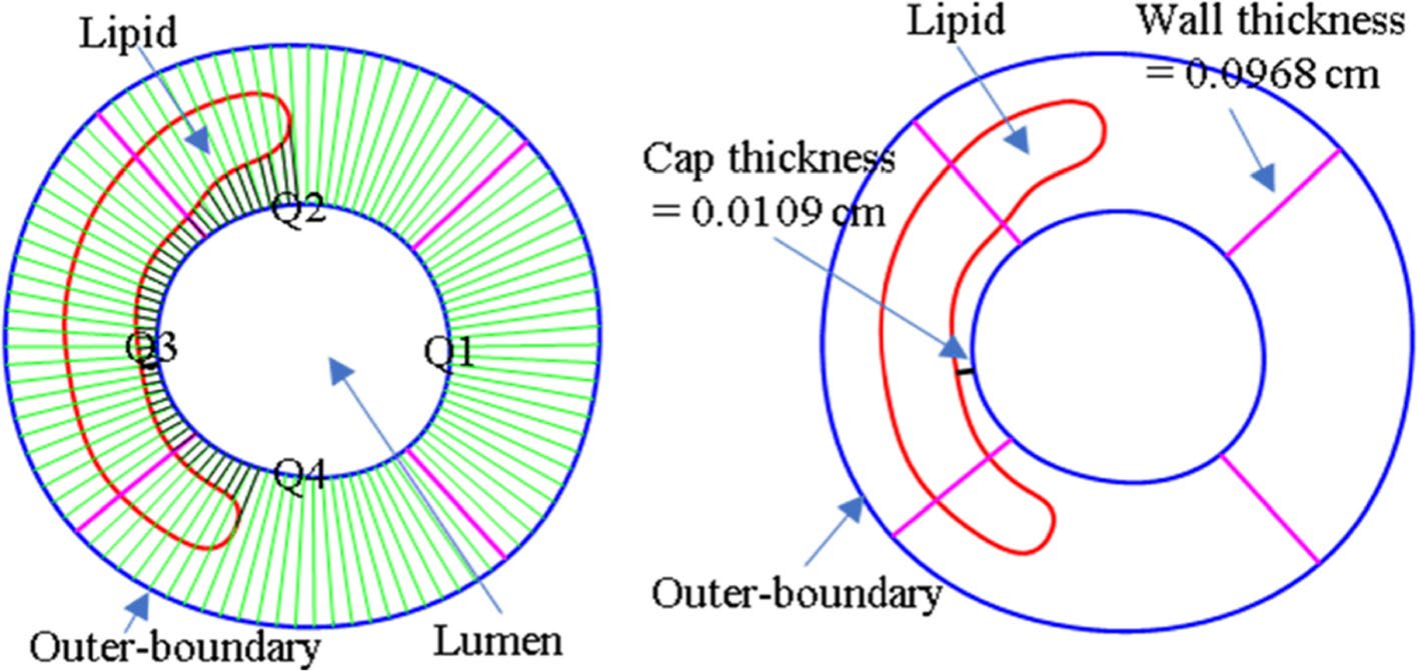
Sketch showing definitions of quarters, wall thickness, cap thickness, and lipid using a four-quarter-even-spacing method

To extract PWS, PWSn and FSS at the inner boundary of the vessel (the interface between fluid and vessel), each slice was divided into 100 points using a 4-quarter-even-spacing method (See Fig. 5) (Guo et al. 2019; Lv et al. 2020, 2023) and FSI model solution results were extracted from those points for analysis. Plaque stress, strain and FSS values were first extracted from each slice (100 data points from each slice lumen). Then the maximum and average of those values over all slices from the 3D model were used for comparison and prediction.

The relative difference of a quantity between plaques with and without erosion was calculated using the following formula:

16 $$ {\text{Relative Difference}} = \frac{{{\text{Results from plaques with erosion}} - {\text{Results from plaques without erosion}}}}{{\text{Results from plaques without erosion}}} \times 100\% , $$

In addition to comparative analysis of plaques with and without erosion, sites with and without erosion in the eroded plaques were also compared to investigate the biomechanical characteristics of locations where erosion tends to happen. As the lumen of each slice was divided into 100 points, each point was labeled as erosion or non-erosion point based on the thrombus information visible in OCT frames (see Fig. 2). Results of PWS/PWSn and FSS in the blood flow at maximum blood pressure were extracted to investigate their differences for erosion and non-erosion locations.

Kolmogorov–Smirnov test was used to test whether the data conforms to a normal distribution and the results indicate that the data did not follow a normal distribution. So, Wilcoxon rank sum test was used to test whether there is a significant difference between eroded and non-eroded data.

### Prediction method for plaque *erosion*

We aim to evaluate the significance of various factors based on their ability to predict plaque erosion status. In potential applications, the prediction method would be applied to plaques without knowing if the plaque had erosion and where the erosion would be. Therefore, we extracted the values of predictor variables from the whole model and used the maximum and mean values for prediction. Our approach includes selecting predictor variables, fitting the prediction model, and evaluating it using the leave-one-out method and bootstrap technique (Hastie et al. 2008). Specifically, we analyzed up to three of the thirteen predictor variables (maximum PWS, mean PWS, maximum PWSn, mean PWSn, maximum FSS, mean FSS, maximum ΔFSS, mean ΔFSS, mean Plaque burden, stenosis severity (by area), minimum cap thickness, mean cap thickness, mean lumen area), resulting in a range of models for analysis: 13 single-predictor models, 78 pairwise-predictor models, and 286 triple-predictor models. We utilized a logistic regression model to fit the data and predict the binary status of plaque erosion, under the assumption that all patients in the study are independent. Our prediction process involved two primary methods. First, the leave-one-out prediction method was applied, using data from fifteen patients for training and one patient for testing, repeated across sixteen possible data splits. This method was preferred over 2- or fivefold cross-validation due to our dataset’s small size, allowing a larger training set and thus a more stable predictive model. We employed the Area Under the Curve (AUC) value as the criterion for prediction accuracy. Second, to determine the robustness and reliability of the predictive models, we conducted 100 bootstrap random-sample simulations and made predictions in each. This procedure enabled us to assess prediction uncertainty, for which we obtained the 90% bootstrap confidence interval of the AUC value.

## Results

Three basic questions for eroded plaque research are: a) what risk factors are the best predictors for plaque erosion; b) what locations and under what conditions erosions may happen; c) what kind of plaques may have (or tend to have) erosion. Details are given below.

### Combination of multiple risk factors led to better prediction accuracies

**Fig. 6:**
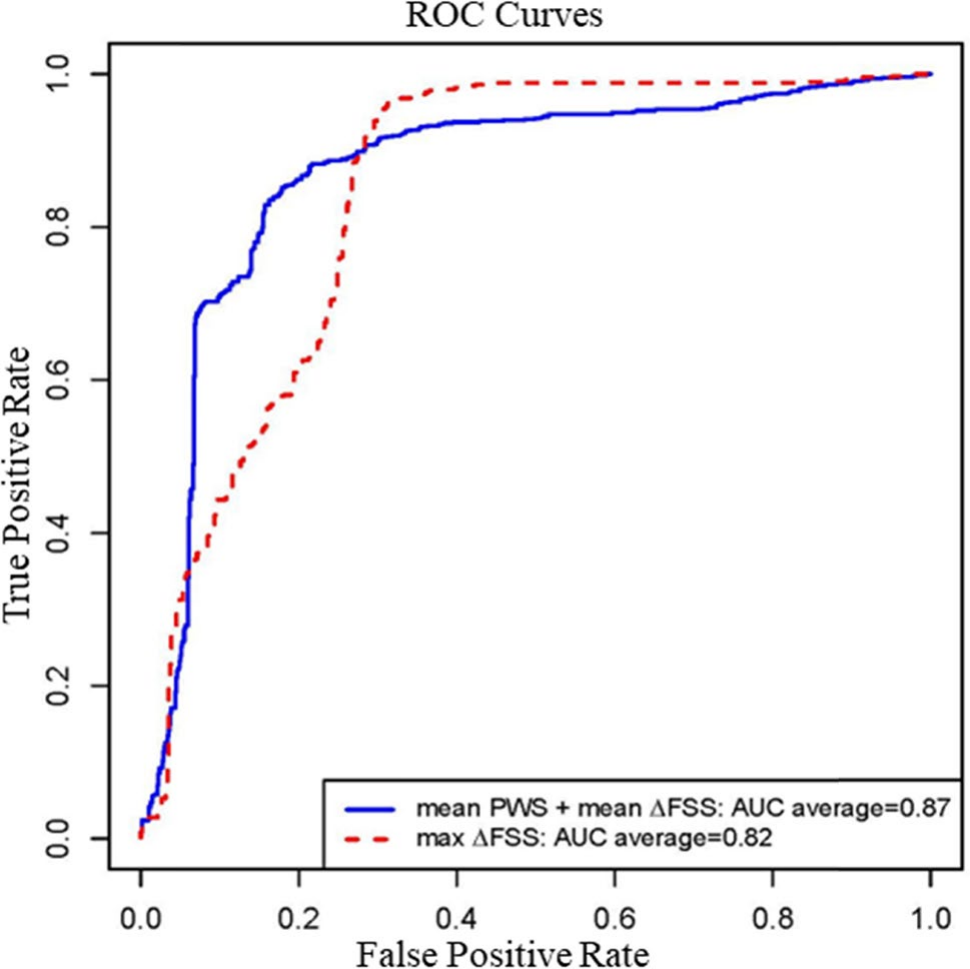
The ROC curves for the top multi-factor model (mean PWS + mean ΔFSS; AUC average = 0.866) and the top single-factor model (max Δ FSS; AUC average = 0.819) based on the cumulated prediction outcomes from all 100 bootstrap random-sample simulations

Table 4 shows the AUC average on 100 rounds of bootstrap random-sample simulations showing combination of multiple risk factors gave better prediction accuracies. To ensure the stability of model fitting for prediction, we apply a conservative strategy to exclude multi-predictor models that contain any significantly correlated predictors (based on a Pearson’s correlation p value < 0.05). The best prediction AUC (area under the curve) was achieved by a combination of mean PWS and mean ΔFSS with AUC average = 0.866, 90% confidence interval (0.717, 1.0). The max ΔFSS was the best single predictor among the 13 predictors considered, with AUC average = 0.819, 90% confidence interval (0.624, 1.0). The ROC curves for the best combination and single predictors based on the cumulated prediction outcomes from all 100 bootstrap random-sample simulations are given by Fig. 6. Table 4 provides some initial evidence that using FSI models and combining morphological, flow shear stress and plaque stress/strain factors may give better prediction accuracies than using single predictors.

**Table 4:** AUC average and 90% confidence interval based on 100 rounds of bootstrap random-sample simulations show the combination of multiple risk factors gave better prediction accuracies

Risk factors	AUC average	90% Confidence interval
The best ten predictors (Single or multi-predictor combinations)		
Mean PWS + mean ΔFSS	0.866	(0.717,1.000)
Mean PWSn + max ΔFSS	0.865	(0.686,1.000)
Max PWS + max ΔFSS	0.822	(0.655,1.000)
Max PWSn + max ΔFSS + Area Stenosis	0.822	(0.607,1.000)
Max ΔFSS	0.819	(0.624,1.000)
Mean PWS + mean ΔFSS + mean Lumen Area	0.818	(0.624,1.000)
Mean PWSn + max ΔFSS + mean Plaque Burden	0.809	(0.624,1.000)
Mean PWSn + mean ΔFSS	0.803	(0.547,1.000)
Mean PWSn + mean ΔFSS + mean Plaque Burden	0.803	(0.563,1.000)
Max ΔFSS + mean Plaque Burden	0.801	(0.546,1.000)
The single predictors		
Max ΔFSS	0.819	(0.624, 1.000)
Max FSS	0.726	(0.453, 0.875)
Mean ΔFSS	0.726	(0.453, 0.875)
Min cap thickness	0.720	(0.515, 0.875)
Mean PWS	0.614	(0.000, 0.875)
Mean PWSn	0.609	(0.000, 0.875)
Mean lumen area	0.604	(0.180, 0.844)
Mean FSS	0.595	(0.312, 0.829)
Area Stenosis	0.586	(0.217, 0.875)
Mean plaque burden	0.502	(0.000, 0.813)
Mean cap thickness	0.431	(0.000, 0.845)
Max PWSn	0.422	(0.000, 0.766)
Max PWS	0.310	(0.000, 0.719)

### Morphological and biomechanical differences for plaques with and without *erosion*

Since cap thickness, stenosis severity (by area) and plaque burden are those most watched risk factors which are closely related to cardiovascular diseases (CVD), Table 5 shows their values for plaques with and without erosion. The average of mean cap thickness (CT) values from plaques with erosion was 309 μm, which was 21.7% thinner than that from plaques without erosion (394 μm). The average of minimum cap thickness values from plaques with erosion was 130 μm, which was 35.9% thinner than that from plaques without erosion (202 μm). The average of stenosis severity (by area) from plaques with erosion was 65.4%, compared to 49.5% from plaques without erosion (*p* = 0.0830). The averages of mean plaque burden from plaques with and without erosion were 53.0% and 49.2%, respectively.

**Table 5:** Morphological feature comparison for plaques with and without erosion

Plaque Type	Patient	Mean Cap Thickness (μm)	Min Cap Thickness (μm)	Stenosis severity by Area (%)	Mean plaque Burden (%)
Plaque with erosion	P1	125	56	69.1	38.8
	P2	397	150	79.8	56.5
	P3	491	304	22.1	38.4
	P4	149	63	72.7	58.7
	P5	249	51	75.4	63.9
	P6	326	130	77.2	42.5
	P7	374	138	69.2	62.6
	P8	360	145	58.1	62.5
	Mean ± SD	309 ± 126	130 ± 82	65.4 ± 18.7	53.0 ± 11.1
Plaque without erosion	P9	293	176	25.3	41.7
	P10	350	234	53.9	48.9
	P11	313	95	68.3	51.5
	P12	255	91	35.6	46.6
	P13	288	190	18.0	48.9
	P14	517	287	50.2	46.7
	P15	486	222	70.4	51.2
	P16	652	324	74.0	57.6
	Mean ± SD	394 ± 141	202 ± 83	49.5 ± 21.3	49.2 ± 4.6
Differences		− 21.7%	− 35.9%	32.3%	7.8%
*P* value		0.5054	0.0830	0.0830	0.4256

Biomechanical plaque stress, strain and flow shear stress may also be closely associated with CVD. Table 6 shows maximum and mean plaque wall stress (PWS) and strain (PWSn), and maximum, mean and minimum flow shear stress (FSS) for plaques with and without erosion. We start from FSS as the differences were clearer. The average of maximum FSS values from plaques with and without erosion were 127.96 dyn/cm^2^ and 72.69 dyn/cm^2^, respectively. The value from eroded plaques was 76.04% higher than that from the non-eroded plaques. The average of mean FSS from plaques with erosion was 61.19% higher than that from the non-eroded plaques (49.24 dyn/cm^2^ vs. and 30.54 dyn/cm^2^). The significance of these differences will be discussed in the Discussion section.

**Table 6:** Plaque wall stress, strain and flow shear stress comparisons for plaques with and without erosion

Plaque Type	Patient	Max PWS (kPa)	Mean PWS (kPa)	Max PWSn	Mean PWSn	Max FSS (dyn/cm^2^)	Mean FSS (dyn/cm^2^)	Min FSS (dyn/cm^2^)
Plaque with erosion	P1	396.8	122.6	0.3724	0.1149	166.97	63.84	9.53
	P2	144.8	63.9	0.1664	0.1048	121.29	47.00	4.95
	P3	289.3	121.6	0.1726	0.1104	86.12	47.20	26.14
	P4	191.1	67.1	0.1827	0.1032	119.73	50.57	8.94
	P5	310.2	77.5	0.1689	0.0974	128.48	50.78	10.30
	P6	182.2	83.3	0.1391	0.0932	96.21	22.29	4.38
	P7	127.0	52.9	0.1542	0.0908	193.46	89.99	16.02
	P8	302.7	76.7	0.2337	0.1147	111.41	22.20	5.65
	Mean ± SD	243.0 ± 95.1	83.2 ± 25.8	0.1988 ± 0.0754	0.1037 ± 0.0093	127.96 ± 35.77	49.24 ± 21.86	10.74 ± 7.27
Plaque without erosion	P9	355.1	136.0	0.1943	0.1288	32.07	16.63	9.57
	P10	238.8	104.1	0.1811	0.1130	96.06	48.59	19.06
	P11	239.1	84.1	0.1786	0.0996	156.35	42.44	12.65
	P12	184.3	96.2	0.1404	0.1129	73.04	36.10	18.60
	P13	323.9	137.8	0.1781	0.1225	117.25	59.81	28.45
	P14	244.1	125.2	0.1661	0.1174	21.16	10.66	3.83
	P15	352.0	100.8	0.1828	0.1081	44.85	19.27	4.90
	P16	171.7	78.5	0.2328	0.1056	40.71	10.84	2.65
	Mean ± SD	263.6 ± 71.9	107.8 ± 22.7	0.1818 ± 0.0260	0.1135 ± 0.0094	72.69 ± 47.26	30.54 ± 18.74	12.46 ± 9.05
Differences		− 7.82%	22.83%	9.34%	− 8.64%	76.04%	61.19%	− 13.82%
P value		0.5737	0.0281	0.7209	0.0830	0.0207	0.0650	0.8785

PWS: plaque wall stress; PWSn: plaque wall strain; FSS: flow shear stress

The plaque stress/strain differences between the two groups were much smaller. The average of maximum PWS from plaques with and without erosion were 243.0 kPa and 263.6 kPa, respectively, with a very small difference of -7.82%. The average of mean PWS from plaques with erosion was 22.83% lower than that for plaques without erosion (83.2 kPa vs. 107.8 kPa). The average of maximum PWSn from plaques with erosion was 9.34% higher than that from plaques without erosion (0.1988 vs. 0.1818). The averages of mean PWSn from plaques with and without erosion were 0.1037 and 0.1135, with a small 8.64% difference.

**Fig. 7:**
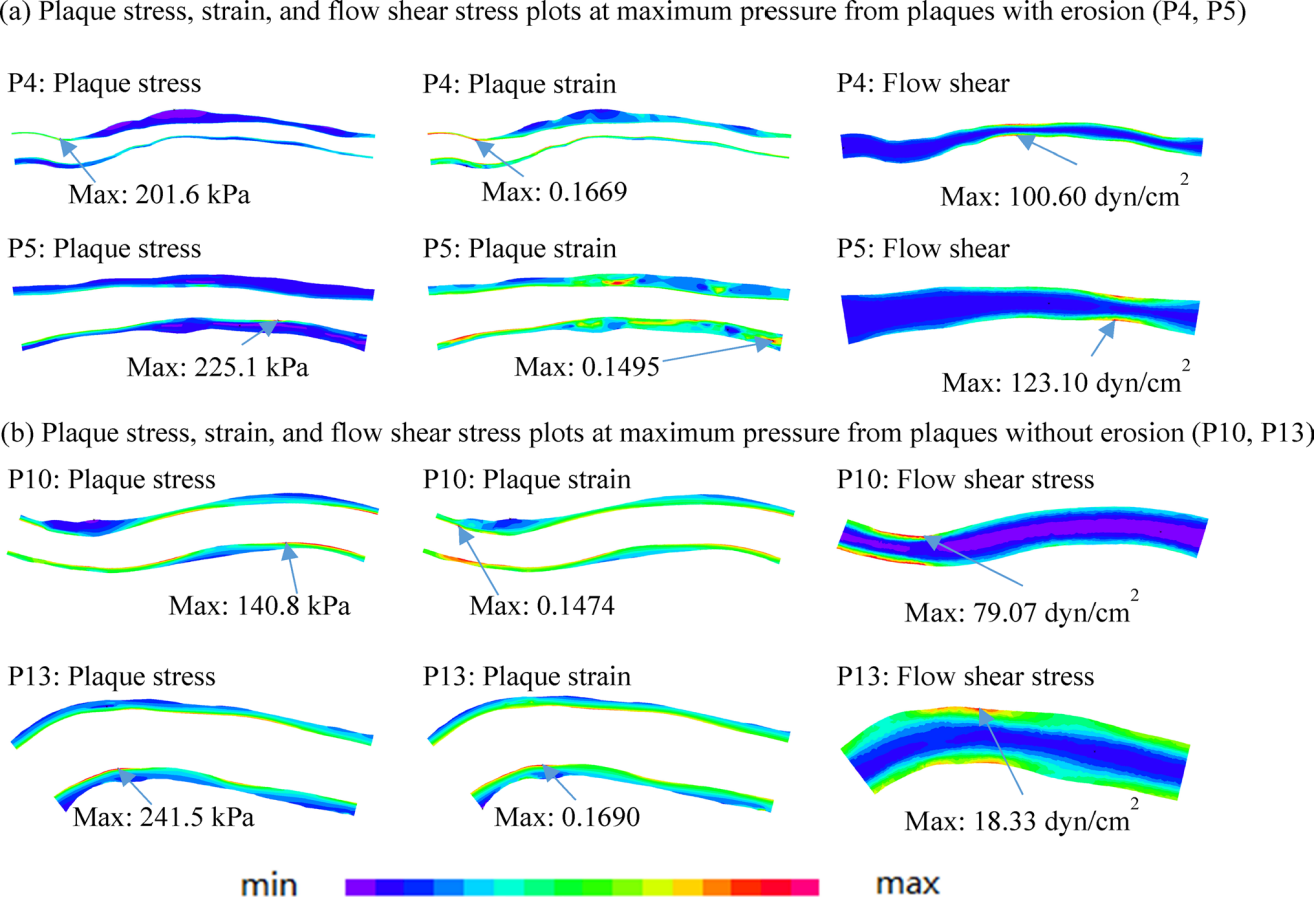
Plaque stress, strain, and flow shear stress plots at maximum pressure from P4, P5, P10 and P13 showing their differences between plaques with and without erosion. P4 and P5: plaques with erosion; P10 and P13: plaques without erosion

It should be noted that the difference of FSS (maximum, mean and min values) between plaques with and without erosion were larger than the corresponding differences of PWS and PWSn (maximum and mean values). Figure 7 provides plaque stress, strain, and flow shear stress plots from eroded plaque (P4, P5) and non-eroded plaques (P10, P13) showing their differences. It is a demonstration that plaque stress and strain did not have much difference between eroded and non-eroded plaques, while FSS showed clear differences between the two groups. We would like to point out that figures do not provide enough detailed information. For comparison purposes, data should be extracted from all usable nodes to give accurate comparisons.

### Maximum, mean and minimum flow shear stress values were higher at *erosion* site

Another focus for erosion study is to find locations where erosion is more likely to occur. To find out biomechanical characteristics of erosion sites, maximum, mean and minimum flow shear stress (FSS) for sites with and without erosion were extracted from those eroded plaques and compared. The results are summarized in Table 7.

**Table 7:** Flow shear stress comparisons for sites with and without erosion

	Patient	Sites with erosion	Sites without erosion	Difference
Max FSS (dyn/cm^2^) P value = 0.1094	P1	158.14	166.97	− 5.3%
	P2	53.22	121.29	− 56.1%
	P3	33.12	86.12	− 61.5%
	P4	119.73	119.54	0.2%
	P5	128.48	128.32	0.1%
	P6	96.21	93.27	3.2%
	P7	173.34	193.46	− 10.4%
	P8	100.08	111.41	− 10.2%
	Mean ± SD	107.79 ± 48.03	127.55 ± 36.16	− 15.5%
Min FSS (dyn/cm^2^) *P value* = 0.0078	P1	114.18	9.53	1097.7%
	P2	51.58	4.95	941.9%
	P3	32.75	26.14	25.3%
	P4	41.97	8.94	369.5%
	P5	77.40	10.30	651.5%
	P6	7.34	4.38	67.5%
	P7	49.53	16.02	209.2%
	P8	6.41	5.65	13.4%
	Mean ± SD	47.64 ± 35.68	10.74 ± 7.27	343.6%
Mean FSS (dyn/cm^2^) *P value* = 0.0781	P1	130.65	62.82	108.0%
	P2	52.63	46.99	12.0%
	P3	32.94	47.22	− 30.2%
	P4	80.93	48.60	66.5%
	P5	105.19	48.13	118.6%
	P6	54.56	18.90	188.6%
	P7	94.18	89.82	4.8%
	P8	21.06	22.43	− 6.1%
	Mean ± SD	71.52 ± 37.63	48.11 ± 22.27	48.6%

FSS: flow shear stress

The average of mean FSS from erosion sites of the eight eroded plaques was 71.52 dyn/cm^2^, which is 48.6% higher than that from sites without erosion (48.11 dyn/cm^2^). The average of minimum FSS from erosion and non-erosion sites of the eight eroded plaques were 47.64 dyn/cm^2^ and 10.74 dyn/cm^2^, respectively. The average of maximum FSS from erosion sites of the eight eroded plaques 15.5% lower than that from sites without erosion (107.79 dyn/cm^2^ vs. 127.55 dyn/cm^2^).

**Fig. 8:**
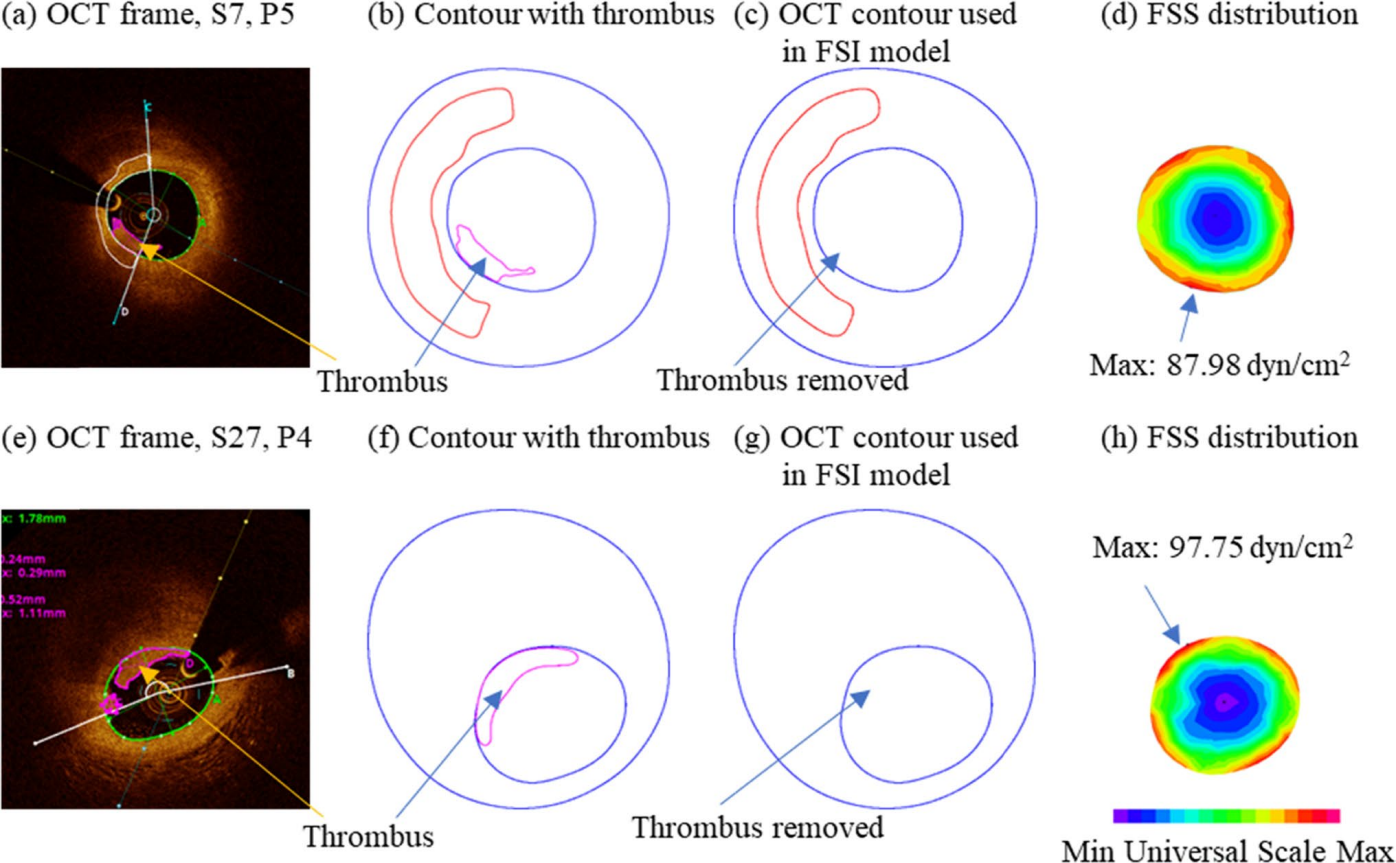
Sample flow shear stress plots at maximum pressure from eroded plaques P4 and P5 showing FSS were higher at eroded locations. FSS: flow shear stress

Combining the above, we could say that erosion sites had higher mean, minimum and maximum FSS than the no-erosion sites, while results from maximum FSS comparison were the weakest. Since maximum and minimum are only one-point data, the mean FSS results should be more reliable. Figure 8 shows two sample flow shear stress plots from eroded plaques P4 and P5 showing that FSS were higher at erosion locations.

### Plaque stress/strain behaviors at *erosion* sites

Table 8 compares maximum and mean plaque wall stress/strain for sites with and without erosion. The average of maximum PWS from erosion sites of the eight plaques with erosion was 131.7 kPa, which is 45.8% lower than that from sites without erosion (243.0 kPa). All eight plaques had lower maximum PWS at erosion sites. The average of maximum PWSn from sites with erosion was 0.1336, which is 32.8% lower than that from sites without erosion (0.1988). All eight cases had lower maximum PWSn at erosion sites.

**Table 8:** Plaque wall stress and strain comparisons for sites with and without erosion

	Patient	Sites with erosion	Sites without erosion	Difference
Max PWS (kPa) *P value* = 0.0078	P1	286.9	396.8	− 27.7%
	P2	80.1	144.8	− 44.7%
	P3	168.2	289.3	− 41.9%
	P4	93.1	191.1	− 51.3%
	P5	165.1	310.2	− 46.8%
	P6	76.9	182.2	− 57.8%
	P7	70.2	127.0	− 44.7%
	P8	113.0	302.7	− 62.7%
	Mean ± SD	131.7 ± 73.5	243.0 ± 95.1	− 45.8%
Mean PWS (kPa) *P* value = 0.9453	P1	151.6	122.2	24.0%
	P2	79.0	63.9	23.7%
	P3	152.8	121.5	25.8%
	P4	56.8	67.7	− 16.2%
	P5	67.4	78.0	− 13.7%
	P6	40.2	87.8	− 54.2%
	P7	41.6	53.4	− 22.0%
	P8	62.1	79.6	− 22.0%
	Mean ± SD	81.4 ± 45.5	84.3 ± 25.5	− 3.4%
Max PWSn *P* value = 0.0078	P1	0.1704	0.3724	− 54.2%
	P2	0.1200	0.1664	− 27.9%
	P3	0.1283	0.1726	− 25.7%
	P4	0.1292	0.1827	− 29.3%
	P5	0.1366	0.1689	− 19.1%
	P6	0.0963	0.1391	− 30.8%
	P7	0.1215	0.1542	− 21.2%
	P8	0.1666	0.2337	− 28.7%
	Mean ± SD	0.1336 ± 0.0246	0.1988 ± 0.0754	− 32.8%
Mean PWSn *P* value = 0.8438	P1	0.1290	0.1147	12.5%
	P2	0.1176	0.1048	12.2%
	P3	0.1220	0.1104	10.5%
	P4	0.0907	0.1040	− 12.8%
	P5	0.0941	0.0976	− 3.5%
	P6	0.0759	0.0950	− 20.1%
	P7	0.0844	0.0911	− 7.4%
	P8	0.1108	0.1155	− 4.1%
	Mean ± SD	0.1031 ± 0.0194	0.1041 ± 0.0091	− 1.0%

Comparison of mean PWS and PWSn had different situation. The average of mean PWS from sites with erosion was only 3.4% lower than that from sites without erosion (81.4 kPa vs. 84.3 kPa). Not only that, among the eight plaques, three cases had higher mean PWS at erosion sites (P1: 24.0%; P2: 23.7%; P3: 25.8%), while the five cases had lower mean PWS at erosion sites (P4: − 16.2%; P5: − 13.7%; P6: − 54.2%; P7: − 22.0%; P8: − 22.0%). Similarly, the average of mean PWSn from sites with erosion of the eight plaques was 1.0% lower than that from sites without erosion (0.1031 vs. 0.1041). Among the eight cases, three cases had higher mean PWSn at erosion sites (P1: 12.5%; P2: 12.2%; P3: 10.5%), while the other five cases had lower mean PWSn at erosion sites (P4: − 12.8%; P5: − 3.5%; P6: − 20.1%; P7: − 7.4%; P8: − 4.1%). Therefore, the mean PWS/PWSn comparisons for sites with and without erosion were inconclusive.

## Discussion

This is a study for plaques with erosion using a relatively larger patient size compared to previous studies (Campbell et al. 2013; Wang et al. 2021). This is due to the difficulty in getting eroded plaque samples. With eight eroded plaques versus eight non-eroded plaques, results could be affected by a single case. Effort was made to randomly select our samples. This should be considered as a pilot study. Results presented could be considered as preliminary and further validations are needed to get more definite conclusions.

Our study was divided into three parts: erosion plaque identification using single and combined predictors, eroded vs. non-eroded plaque comparisons and eroded location vs. non-eroded location comparisons within plaques with erosions. The following discussions will focus on biomechanical differences as those are the purpose of our modeling study.

### Removing *thrombus* to obtain approximated pre-*erosion* plaque morphology for model construction and biomechanical calculations

It is of great interest and importance to identify plaques which could potentially develop erosion. However, pre-erosion OCT data is generally not available. Using the plaque data with thrombus removed is arguably the best possible pre-erosion data we can get to construct biomechanical models. With those data, we could compare biomechanical factors for plaques with and without erosion on somewhat equal “pre-erosion” ground. The thrombus in plaques with erosion is mainly white thrombus. OCT can “see” the thrombus and the lumen line behind it. Figure 1 shows both thrombus and the lumen line behind it clearly. So it is easy to remove the thrombus from the segmented slices and recover the lumen line to obtain the pre-erosion lumen. The flow dynamics for plaques with thrombus is very different since thrombus blocks blow flow and it is not possible to have a fair comparison of flow behaviors (Wang et al. 2021).

### Combining multiple risk factors may give better identification/prediction accuracy for plaque *erosion*

Identification or prediction of plaques which are more likely to develop erosion is of clear clinical significance. By using reconstructed pre-erosion plaque morphologies and FSI models, we were able to obtain plaque pre-erosion plaque morphology, flow shear stress and plaque stress and strain conditions. At the same time, the real plaque erosion cases (the “future” event relative to the pre-erosion models) served as gold standard for prediction models and their predictions outcomes. Our results indicated that the max ΔFSS was the best single predictor with AUC average = 0.819, 90% confidence interval (0.624, 1.0), while the best prediction was achieved by a combination of mean PWS and mean ΔFSS with AUC average = 0.866, 90% confidence interval (0.717, 1.0). This is a 5.7% improvement in AUC value. This pilot result suggested that plaque stress/strain and flow shear stress complemented each other and led to prediction improvement.

### Higher flow shear stress may be associated with plaque *erosion*

Injury to the endothelium is one of the initial factors of plaque erosion and flow shear stress is considered as an important driver of PE (Staarmann et al. 2019). Previous studies suggested that higher flow shear stress plays an important role in the progression of PE (Campbell et al. 2013; Thondapu et al. 2021; Wang et al. 2021). Our results showed that the average of maximum FSS values from eroded plaques was 127.96 dyn/cm^2^, 76.04% higher than that from the non-eroded plaques, and the average of mean FSS from plaques was 61.19% higher than that from the non-eroded plaques (49.24 dyn/cm^2^ vs. and 30.54 dyn/cm^2^). These results are consistent with the findings in (Campbell et al. 2013; McElroy et al. 2021). It is worth noting that models in (Campbell et al. 2013; McElroy et al. 2021; Wang et al. 2021) included thrombi which would narrow the lumen, increase stenosis severity, and lead to higher flow and flow shear stresses. Our models had thrombi removed. However, we still obtained higher flow shear stress values for eroded plaques. This is a good reinforcement of the suggestive claim that “higher flow shear stress may be associated with plaque erosion”.

### Plaque wall stress and strain had smaller differences between eroded and non-eroded plaques

Unlike the flow shear stress situation for eroded/non-eroded plaque comparisons, the average of maximum PWS from plaques with erosion was only 7.82% lower than that from plaques without erosion and the average of mean PWS from plaques without erosion is 22.83% lower than that from plaques without erosion. The situation for plaque strain was even harder to read: the average of maximum PWSn from plaques with erosion is 9.34% higher than that from plaques without erosion and the average of mean PWSn from plaques without erosion is 8.64% lower than that from plaques without erosion. The results show that it is hard to draw any conclusion for the impact of PWS and PWSn on plaque erosion. Larger patient size studies are needed to get a clearer picture, if one exists.

### *Erosion* locations were associated with high flow shear stress conditions

For erosion locations, our results showed that the average of mean FSS from sites with erosion was 48.6% higher than that from sites without erosion. The average of minimum FSS from sites with erosion was 343.6% higher than that from sites without erosion. With these results, it could be seen that erosion sites were associated with higher flow shear stress conditions (mean and minimum).

We also had the results that the average of maximum FSS from sites with erosion was 15.5% lower than that from sites without erosion. This is considered less important than the differences in mean and minimum FSS because a) the percentage is lower; b) maximum value is only a one-note value while mean value is the average of all erosion notes.

### *Erosion* locations had lower maximum plaque wall stress and lower maximum plaque strain

When comparing erosion sites with non-erosion sites, we did find that the average of maximum PWS from erosion sites of the eight plaques with erosion was 131.7 kPa, which is 45.8% lower than that from sites without erosion (243.0 kPa). All eight plaques had lower maximum PWS at erosion sites. The average of maximum PWSn from sites with erosion was 0.1336, which is 32.8% lower than that from sites without erosion (0.1988). All eight cases had lower maximum PWSn at erosion sites.

Other than these, other plaque wall stress and strain values had mixed pictures. For example, the average of mean PWS from sites with erosion was 81.4 kPa, which is 3.4% lower than that from sites without erosion (84.3 kPa). However, among the eight plaques, three cases had higher mean PWS at erosion sites, while the five cases had lower mean PWS at erosion sites. The average of mean PWSn from sites with erosion of the eight plaques was 0.1031, which is only 1.0% lower than that from sites without erosion (0.1041). The behaviors of the eight cases were not uniform: three cases had higher mean PWSn at erosion sites, five cases had lower mean PWSn at erosion sites.

### Method clarification, result interpretation, and limitation of small data size

We would like to clarify our methods and the interpretation of the results within the context of a limited sample size. To address potential model overfitting, we carefully followed the classic leave-one-out cross-validation procedure. Model overfitting occurs when the statistical model performs well on training data but poorly on testing data (Montesinos Lopez et al. 2022). For the given data, the leave-one-out procedure iteratively splitting the data into different training and one-subject testing datasets, resulting a more balanced overall prediction accuracy against potential overfitted models.

We also recognize that our predictive factors may be correlated, which could lead to inaccurate estimates of the effects of individual factors. However, our focus is on the combined influence of these factors on the prediction, which is more tolerant of correlations among the predictors.

We acknowledge the limitation of our current data due to its small size. Consequently, the measured AUC is specific to the given dataset, and we caution against over-extrapolating it as a population-level prediction accuracy. Overall, with the data at hand, the main takeaway is that combining appropriate predictive factors can provide better predictions than using the factors individually. However, the exact predictive accuracy on a larger scale at the population level remains to be studied using larger datasets.

### Other limitations

We have the following limitations: a) The study database was small due to the difficulty of getting eroded plaque samples and time requirement to construct 3D FSI models; b) Patient-specific vessel material properties were not available. So parameter values in the current literature were used in our models; c) Vessel side branches were neglected because flow data for those branches were not available; d) Adventitia was not included in the vessel model due to penetration limitation of OCT; e) coronary cyclic bending due to heart contract was not included to save time; f) Opening angle and residual stress were not included in our models. For 3D curved vessels, including residual stress (circumferential) remains to be a challenge. We did include axial shrink and stretch and radial shrink and pressurization which are actually residual stress in the axial and radial directions.

## Conclusion

This is a study for plaque erosion using OCT-based full 3D FSI models and a relatively larger patient size compared to previous studies (Campbell et al. 2013; Wang et al. 2021). Our FSI models were constructed with thrombosis removed so that the model results (especially FSS) could reflect the biomechanical environment of the plaque before erosion started to develop. Our results suggested that combining multiple risk factors may give better prediction accuracies for plaque erosion. In terms of comparisons, flow shear stress had greater differences between erosion and non-eroded plaques than plaque wall stress and strain conditions. This is a pilot study, and large-scale patient studies are needed for further improvements and validations.

## Data Availability

All data, models, or code generated or used during the study are available from the corresponding author upon reasonable request.
